# Laxative Activity of the Hot-Water Extract Mixture of *Hovenia dulcis* Thunb. and *Phyllostachys pubescens* Mazel in Chronic Constipation Model SD Rats

**DOI:** 10.4014/jmb.1911.11051

**Published:** 2020-02-18

**Authors:** Kyo-Nyeo Oh, Yujin Kim, Eun Jin Choi, Hyunmi Lee, Ji Ae Hong, Miri Kim, Dool-Ri Oh, Myung-A Jung, Ro-Dong Park, Seong-il Kim, Ju-seon Yong, Hui-Seop Lee, SangOh Ban, Chul-yung Choi

**Affiliations:** 1 Jeonnam Bioindustry Foundation, Jeonnam Institute of Natural Resources Research, Jangheung-gun 59338, Republic of Korea; 2 Agroceuticals Lab, Haenam Natural Farming Association Corporation, Gwangju 61111, Republic of Korea

**Keywords:** Constipation, *Hovenia dulcis* Thunb., laxative activity, Ca^2+^ influx, *Phyllostachys pubescens* Mazel

## Abstract

This study examined the laxative effects of hot-water extracts of *Hovenia dulcis* Thunb. (HD), *Phyllostachys pubescens* Mazel (PM), and a 2:8 mixture of both (HP) in two chronic constipation models. For the loperamide-induced constipation model, animals were divided into an untreated group, negative control group (loperamide 4 mg/kg), positive control group (bisacodyl 4 mg/kg) group, and six treatment groups (HP 100 or 400, HD 50 or 100, and PM 100 or 400 mg/kg). For the lowfiber diet-induced constipation model, animals were divided into an untreated group (normal diet), negative control group (low-fiber diet), positive control group (Agio granule, 620 mg/kg), and the same treatment groups. Fecal number, weight, fecal water content, and intestinal transit ratio were higher in the groups treated with HP, HD, and PM than in the groups treated with loperamide or lowfiber diet. Thickness of colon mucosa and muscle layers were increased in the treated groups. Colon tension increased in the HP groups, and [Ca^2+^]i measurements using fura-2 as an indicator showed that HP inhibits ATP-mediated Ca^2+^ influx in IEC-18 cells. These results showed that the HP mixture has laxative activity by increased mucin secretion and inducing contractile activity and relaxation. It may be a useful therapeutic strategy for ameliorating in chronic constipation.

## Introduction

Gastrointestinal dysfunction is characterized by various symptoms including abdominal inflation, constipation, diarrhea, discomfort, anxiety, or depression [1]. Chronic constipation is one of the most common gastrointestinal issues, affecting about 15% of all adults and 30% of those over the age of 60 [2]. Constipation is characterized by infrequent or difficult evacuation of feces, defined as fewer than three bowel movements per week [3, 4]. Currently, various laxatives are used to decrease symptoms and most solutions for constipation focus on modulating the motility of the gastrointestinal tract [5]. The laxatives for constipation treatment are bulk-forming agents (*i.e.* bran, seaweed, methylcellulose derivatives, and calcium polycarbophil), osmotic agents (*i.e.* magnesium salts and sodium phosphate), hyperosmotic agents (*i.e.* lactulose, sorbitol, lactitol, polyethylene glycol, stimulant agents (*i.e.* senna, aloe, and bisacodyl), surfactants (*i.e.* docusate, castor oil and dehydrocholic acid), and other laxatives (*i.e.* 5-hydroxytryptamine (5-HT) 4 agonists, Cl-channel activators, and guanylate cyclase-C agonists) [6]. Each agent has one or more mechanisms for constipation treatment. However, some of these drugs are reported to have severe side effects (cisapride as promotility agent: cardiac arrhythmias [7, 8]; tegaserod as selective 5-HT_4_ receptor agonist [9]: coronary artery contraction, coronary spasm, and myocardial infarction). Moreover, long-term treatment with stimulant laxatives leads to steatorrhea, pancreatitis, renal failure, rhabdomyolysis, and melanosis coli [10-13.]. Many studies, including clinical trials, have been carried out to develop new safer therapies for chronic constipation [14].

*Hovenia dulcis* Thunb., of the Rhamnaceae family, is a hardy tree found in Korea, Japan, Eastern China, and other parts of Asia [15]. The roots, seed, fruit, leaves, and branches of *Hovenia dulcis* Thunb. have been used as a traditional herbal medicine. It is well-known for treating liver diseases, alcohol-induced hangover, cancer, steatosis, inflammation, acute hyperlipidemia, and atopic dermatitis-like skin lesions [16-21]. Although many studies have revealed the medical efficacy of *Hovenia dulcis* Thunb., there have been very few studies focusing on alleviating constipation.

Bamboo, of which there are approximately 75 species, is a common plant in Asia. There are 200 varieties of *Phyllostachys* including *Phyllostachys pubescens* Mazel (Moso bamboo), *Phyllostachys nigra*, *Sasa borealis*, *Phyllostachys bambusoides*, and *Phyllostachys nigra* var. *henonis* (Mitford) Stapf. ex Rendle [22]. Bamboo extracts have diverse physiological functions such as antioxidant, free radical-scavenging, antiobesity, anticancer, neuroprotective, immune regulating, and antifungal effects [22-26]. *Bambusae caulis*, a medicinal herb derived from the inner branch of *Phyllostachys nigra* var. *henonis* (Mitford) Stapf. ex Rendle, has been used to treat inflammation, fever, diarrhea, and intestinal inflammation [27-29].

As a preliminary experiment, constipation relief experiments were performed with various ratios (2:8, 5:5, and 8:2) of *Hovenia dulcis* Thunb. (HD) branch and *Phyllostachys pubescens* Mazel (PM) hot water extracts. The HD and PM 2:8 mixture (HP 2:8 mixture) was determined to be the most effective for improving constipation, and the mechanism of constipation alleviation was investigated.

In this study, we evaluated the laxative effect of the extract of *Hovenia dulcis* Thunb., *Phyllostachys pubescens* Mazel, and a mixture of the two. We found out that the extract of mixture of the two (HP 2:8) showed a laxative effect when treating loperamide-treated constipation model rats and low-fiber diet-treated constipation model rats. The HP 2:8 mixture’s mechanism of alleviating constipation of has been elucidated for further application.

## Materials and Methods

### Chemicals

Loperamide hydrochloride, gum arabic, charcoal, hematoxylin & eosin, and the voltage-dependent Ca^2+^ channel (VDCC) blocker nifedipine were purchased from Sigma Chemical Co. (USA). Dulcolax S was purchased from Sanofi-Aventis Korea Co. (Korea). Agio granule was purchased from BUKWANG PHARM.CO.,LTD (Korea). IEC-18 (ATCC CRL-1589), an epithelial cell line derived from the colon of rat intestines, was purchased from American Type Culture Collection (USA). Dulbecco’s Modified Eagle Medium (DMEM) and Fetal Bovine Serum (FBS) were purchased from Invitrogen Inc. (USA).

### Plant Material

The branches of *Hovenia dulcis* Thunb. (HD) were purchased from Jangheung Heotgae Farming Association Corporation, Jangheung-Gun (Jeollanam-Do) and the leaves of *Phyllostachys pubescens* Mazel (PM) from Baekok Industry, Gyeongsan (Gyeongsangbuk-Do) in South Korea. Air-dried branches and leaves were chopped into small pieces. The chopped branches (20 kg) of HD were extracted twice with 200 L of water at 100 ± 5°C for 3 h and combined together. The combined extract was concentrated at 85°C for 5 h, lyophilized, pulverized and passed through a 60 mesh sieve. The final yield of HD was 3.7%. The chopped leaves (20 kg) of PM were extracted 2 times with 400 L of water at 100 ± 5°C for 3 h, and concentrated, lyophilized, and pulverized under the same process as for HD. The yield of PM was 12.9%. *Hovenia dulcis* Thunb. (HD) and *Phyllostachys pubescens* Mazel (PM) mixed (2:8) hot-water extract (HP 2:8 mixture) was prepared with 200 g of HD and 800 g of PM. The same samples as those used in the clinical trial study which is approved by Institutional Review Board (IRB), were used for present study. IRB approval: Institutional Review Board of Wonkwang University, Gwangju medical center (2018.05.02). Clinical trials registration number: WCTC-HD-PM Ver 1.0.

### Animals

Male Sprague-Dawley rats (6 weeks old) were purchased from Samtako Bio Korea (Korea). Animals were maintained at a constant room temperature of 22 ± 2°C with a humidity level of 50 ± 5% and with free access to water and food under a 12:12 h light : dark cycle (lights on at 8:00 am). The animals were acclimatized for 7 days before beginning the experiments. All experimental procedures were conducted in accordance with the relevant guidelines for the care of experimental animals and were approved by the Institutional Animal Care and Use Committee (IACUC) at the Jeonnam Institute of Natural Resources Research (approval number JINR-1807 and JINR-1823). Every experimental group consisted of 7 animals and each rat was used only once. We randomly assigned seven rats to each of six experimental groups (100 and 400 mg/kg HP 2:8 mixture, 50 and 100 mg/kg HD and 100 and 400 mg/kg PM) and also to a normal control group, loperamide or low-fiber diet group and positive group (bisacodyl, Agio granule). Agio granule is the bulk-forming agent contained the psyllium husk. The loperamide was given for all groups except normal control group. This also applied the low-fiber diet-induced constipation model. Administration of experimental samples for the two constipation rat models began after 1 week of adaptation and observation.

### Experimental design for constipation

**Fig. 1:**
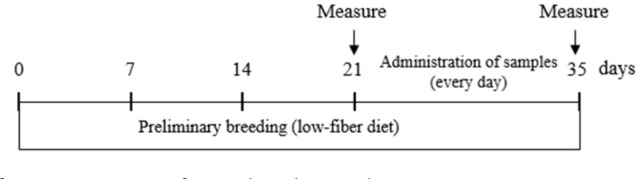
The schedule of examination performed in this study.

For the loperamide-induced constipation model, constipation was induced in the animals through oral administration of 4 mg/kg loperamide hydrochloride daily for 7 days and 1 h prior to experiment sample administration, as described previously [30, 31]. The normal control group was administered saline. The HP 2:8 mixture (100 and 400 mg/kg), HD (50 and 100 mg/kg), and PM (100 and 400 mg/kg) were dissolved in distilled water (DW) and administered orally 1h after loperamide administration for 7 days. The positive control (bisacodyl 4 mg/kg) was dissolved in DW and administered orally as a treatment reference [32]. For the low-fiber diet-induced constipation animal model, the rats were fed a normal or low-fiber diet. The low-fiber diet containing corn starch (41.5%), corn oil (6.0%), dextrin (10.0%), mineral mixture (7.0%), vitamin mixture (1.0%), sucrose (10.0%), and milk casein (24.5%) was purchased from Saeronbio Inc. (Korea) (Table 1). After 21 days of administration of the diet to induce constipation, experimental samples were orally administered for 14 days (Fig. 1). Food intake, water intake, body weight, and fecal water content were measured, and tissue samples were taken from each rat.

### Analysis of Body Weight, Food Intake, and Water Intake

For loperamide-induced constipation, the body weight was measured every two days before administration of loperamide. Food intake and water intake were measured every day for all of the animals before administration of loperamide. For low-fiber diet-induced constipation, the body weight was measured every 7 days, and food intake and water intake were measured daily at 9:00 am.

### Evaluation of Constipation (Fecal Parameters)

Fecal parameters were measured every day for all of the animals before administration. The fecals were collected and dried at 70°C fornd the total water content was calculated as the difference between the wet and dry weights of fecals. Fecal water content (%) = [(fecal wet weight – fecal dry weight) / fecal wet weight] × 100.

### Gastrointestinal Charcoal Transit Ratio and Tissue Sampling

Charcoal excretion containing gum arabic was performed on the last day of the experiment. Measurement of the gastrointestinal propulsion of a charcoal meal was determined according to the modified method by Choi *et al*.[30]. Briefly, the animals were fasted for 18 h prior to the experiment, but were permitted water (*ad libitum*). After 18 h, the animals were orally administrated 1 ml gum arabic with charcoal (5%/10%, w/w) via the same route immediately after sample administration. Gum arabic (5% w/w) was orally administrated as a vehicle. At 30 min after the charcoal meal administration, the animals were anaesthetized. Then, the total intestine length and charcoal meal transit distance were measured and the fecals in the distal colon were counted. The intestinal charcoal transit ratio was expressed as a percentage of travelled distance: Charcoal transit ratio (%) = [(total intestine length - transited charcoal meat distance)/total intestine length] × 100. The colonic segment was isolated with ligatures and fixed with 10% formaldehyde at the time of intestinal charcoal transit ratio measurement.

### Histological Analysis

The fixed tissue segments were embedded in paraffin and serially cut into cross sections 5 µm thick. The sections were stained with H&E. Five tissue segments per group were prepared and the histological profiles interpreted. The colonic mucosal thickness, villus length, crypt thickness and muscle thickness were measured by Leica Application Suite (Leica Microsystem, Switzerland) using a light microscope.

### Preparation of Colon

Male SD rats (200~250 g) were fasted for 18h and sacrificed under anesthesia. An approximately 7 cm length of the colon was removed and placed in Krebs solution containing NaCl 111, KCl 5.9, MgCl_2_ 1.2, CaCl_2_ 2.0, KH_2_PO_4_ 1.2, NaHCO_3_ 25, and glucose 11.5 mM. The Krebs solution was maintained at a pH of 7.4 and continuously aerated with carbogen (95% O_2_/5% CO_2_). Subsequently, the colon was flushed with buffer solution, cleaned of mesenteric tissues and cut into segments 0.5cm in length. The colon segments were clamped in 10 ml organ baths containing Krebs solution maintained at 37°C and aerated with carbogen. Contractions of the longitudinal muscle of isolated colon were measured with force transducers connected to a physiograph recorder (Labchart; AD Instruments, Australia) and a Labchart data-acquisition system. A basal tension of 1 g was applied and allowed to relax, and then repeated tension was applied until the basal tone remained steady at about 0.6 g [33].

### Measurement of Contractile Activity

The colon segments were allowed to equilibrate for a period of 1 h and the Krebs buffer was replaced every 15 min. After 1 h of equilibration, HP 2:8 mixture (0.5, 1, 2 mg/ml) was dissolved in Krebs buffer and tested on each preparation to ensure that a stable and acceptable sensitivity had been reached. The spontaneous contractility of isolated rat colon was measured in the form of average contractile force (in grams over baseline) in a 30-min period. The change in spontaneous contraction (amplitude) was calculated as the average for 1 min, and the frequency of spontaneous contraction was measured over 5 min [34].

### Cell Culture 

The IEC-18 rat ileum cell line was cultured in DMEM supplemented with 10% FBS, 100 U/ml penicillin, and 100 μg/ml streptomycin at 37°C in a humidified atmosphere containing 5% CO_2_.

### Intracellular Calcium Imaging

The acetoxymethyl-ester form of fura-2 (fura-2/AM) was used as the fluorescent Ca^2+^ indicator. According to the modified method by Yang Z *et al*. [35], Cells were incubated for 60 min at room temperature with 5 μM fura-2/AM and 0.001% Pluronic F-127 in normal physiological saline solution (NPSS) composed of (in mM): 137 mM NaCl, 5 mM KCl, 1 mM MgCl_2_, 2.5 mM CaCl_2_, 10 mM HEPES, and 11 mM glucose. The IEC-18 cells were stabilized in NPSS and Ca^2+^-free physiological saline solution (PSS : 137 mM NaCl, 5 mM KCl, 1 mM MgCl_2_, 2 mM *EDTA*, 10 mM HEPES, 11 mM glucose) was treated for 100 s in order to NPSS treated for 300 s. The PSS + HP 2:8 mixture (50, 100, 200 μg/ml) was treated for 100 s and the NPSS + HP 2:8 mixture (50, 100, 200 μg/ml) was treated for 300 s. After that, the NPSS + HP 2:8 mixture (50, 100, 200 μg/ml) + ATP (100 μM) were treated for 300s. Cells were illuminated using a Lambda XL and excitation wavelengths (340 and 380 nm) were selected. Data were acquired every 2 s. All imaging data were collected and analyzed using MetaMorph software.

### Statistical Analysis

All data were expressed as the mean ± standard error of the mean (SEM). The data were statistically evaluated using Student’s *t*-tests or one-way analyses of variance (ANOVA) using GraphPad Prism (GraphPad Inc., USA) software program. The differences between the groups were assessed by Dunnett’s multiple range test. The differences were considered significant at *p* < 0.05.

## Results and Discussion

### Effects on Fecal Parameters in Loperamide-Induced Constipation

**Fig. 2:**
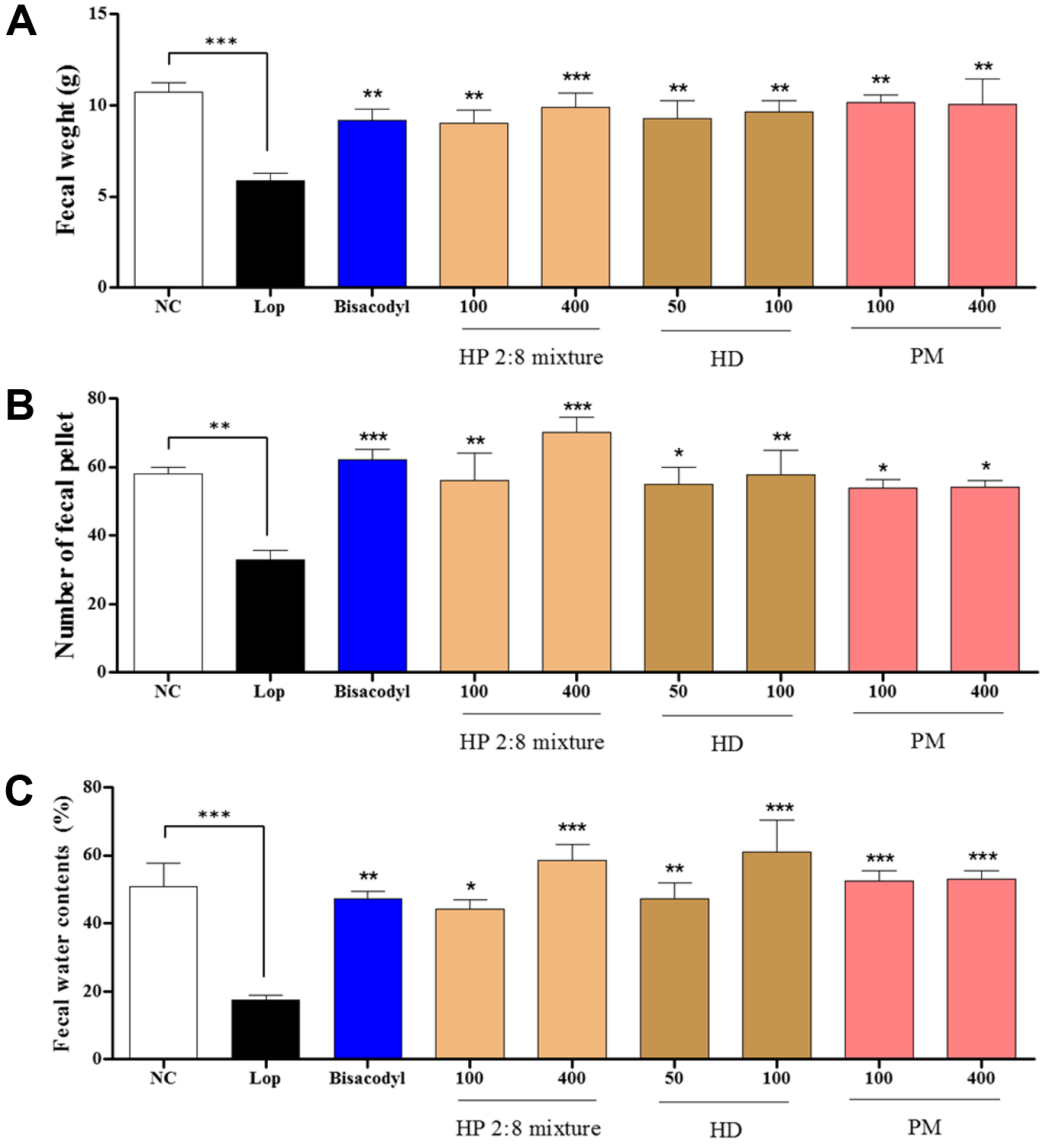
Effect of *Hovenia dulcis* Thunb. (HD) and *Phyllostachys pubescens* Mazel (PM) mixed (2:8) hot-water extract (HP 2:8 mixture) on fecal parameters in loperamide-dosed rats. The effects on (**A**) fecal weight, (**B**) fecal numbers (**C**) fecal water contents in loperamide-induced constipation. The values are expressed as the mean ± SEM (*n* = 7, *p < 0.05, **p < 0.01, ***p < 0.001 significantly fecaldifferent from the loperamide group). Statistical significance was tested with post hoc Dunnett test. NC: normal control, Lop: loperamide treated group, Bisacodyl: positive control, HP: *Hovenia dulcis* Thunb. (HD) and *Phyllostachys pubescens* Mazel (PM) 2:8 mixture.

The fecal weight, number, and water content were assessed to determine the effects of HP 2:8 mixture on loperamide-induced constipation. Fecal weights were increased in HP mixed extract groups (100 and 400 mg/kg) compared to those of the loperamide group and also increased in HD only (100 mg/kg) and PM only (400 mg/kg) groups (Fig. 2A). Fecal numbers were significantly increased by HP mixed extracts at 100 and 400 mg/kg in a dose dependent manner compared to the loperamide group and also increased by HD only at 100 mg/kg and PM only at 400 mg/kg (Fig. 2B). The fecal water content was significantly increased in the groups treated with HP mixed extracts (100 and 400 mg/kg), HD only (50 and 100 mg/kg) and PM only (100 and 400 mg/kg) compared to the loperamide group (Fig. 2C).

### Effect of HP 2:8 Mixture on Body Weight and Feeding Behavior in Loperamide-Induced Constipation

Table 2 shows the results of the analysis of weight changes, food intake, and water intake in each group during the experimental period. Body weight of the loperamide group and the sample treated group were decreased compared with that of the normal control group on the final day, but no abnormal symptoms were observed. Food intake and water intake differed between all groups compared to the control or loperamide group, but no abnormal symptoms were observed. The reason for the decrease in body weight is probably due to the fact that the food intake did not increase significantly.

### Effects on Fecal Parameters in Low-Fiber Diet-Induced Constipation

**Fig. 3:**
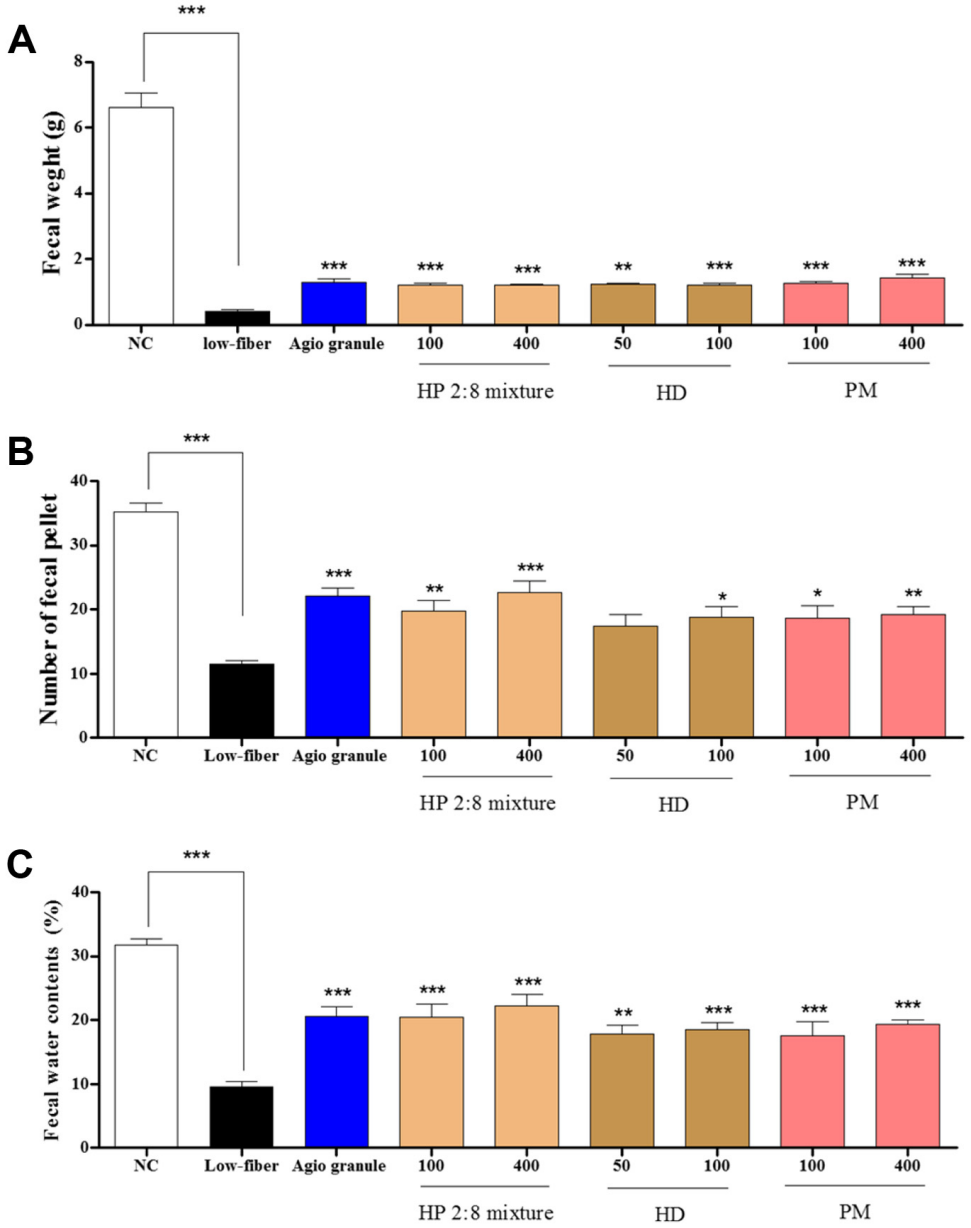
Effect of *Hovenia dulcis* Thunb. (HD) and *Phyllostachys pubescens* Mazel (PM) mixed (2:8) hot-water extract (HP 2:8 mixture) on fecal moisture in low-fiber diet-induced constipation. The effects on (**A**) fecal weight, (**B**) fecal numbers (**C**) fecal water contents in low-fiber diet-induced constipation. The values are expressed as the mean ± SEM (*n* = 7, *p < 0.05, **p < 0.01, ***p < 0.001 significantly different from the low-fiber diet group). Statistical significance was tested with post hoc Dunnett test. NC: normal control, Low-fiber: low-fiber diet group, Agio granule: positive control, HP: *Hovenia dulcis* Thunb. (HD) and *Phyllostachys pubescens* Mazel (PM) 2:8 mixture.

The fecal weight, number, and water content were assessed to determine the effects of HP mixed extracts on low-fiber diet-induced constipation. Fecal weights were significantly increased in HP mixed extract groups (100 and 400 mg/kg) in a dose dependent manner compared to those of the low-fiber diet group and also increased in HD only (100 mg/kg) and PM only (400 mg/kg) groups (Fig. 3A). Fecal numbers were increased by HP mixed extracts at 100 and 400 mg/kg compared to those of the low-fiber diet group and also increased in HD only 100 and PM only 400 mg/kg (Fig. 3B). The water content of fecal was significantly increased by HP mixed extracts (100 and 400 mg/kg), HD only (50 and 100 mg/kg) and PM only (100 and 400 mg/kg) compared to that of the low-fiber diet group (Fig. 3C). Parameters of fecal excretion were increased in all groups that received HP mixed extract, although reduced food intake was observed over the entire experimental period.

### Effect of HP 2:8 Mixture on Body Weight and Feeding Behavior in Low-Fiber Diet-Induced Constipation

Table 3 shows the results of the analysis of weight changes, food intake and water intake in each group during the experimental period. Body weight of the low-fiber diet group and the sample treated group were increased compared with the normal control group from the final day. Food intake and water intake differed between all groups compared to the normal control group, but no abnormal symptoms were observed. The parameter of fecal excretion was increased in all groups that received HP mixed extract, although reduced food intake and water intake were observed over the entire experimental period.

**Fig. 4:**
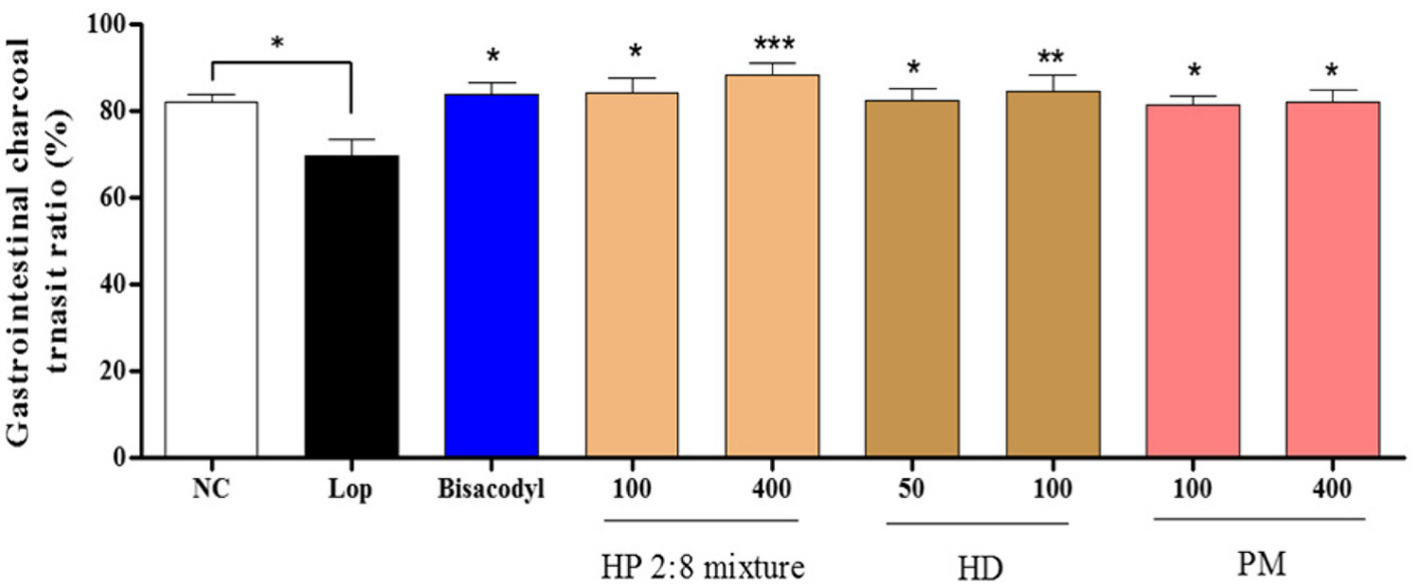
Effect of *Hovenia dulcis* Thunb. (HD) and *Phyllostachys pubescens* Mazel (PM) mixed (2:8) hot-water extract (HP 2:8 mixture) on gastrointestinal charcoal transit ratio in rats with loperamide-induced constipation. The values are expressed as the mean ± SEM (*n* = 7, *p < 0.05, **p < 0.01, ***p < 0.001 significantly different from the loperamide group). Statistical significance was tested with post hoc Dunnett test. NC: normal control, Lop: loperamide treated group, Bisacodyl: positive control, HP: *Hovenia dulcis* Thunb. (HD) and *Phyllostachys pubescens* Mazel (PM) 2:8 mixture.

**Fig. 5:**
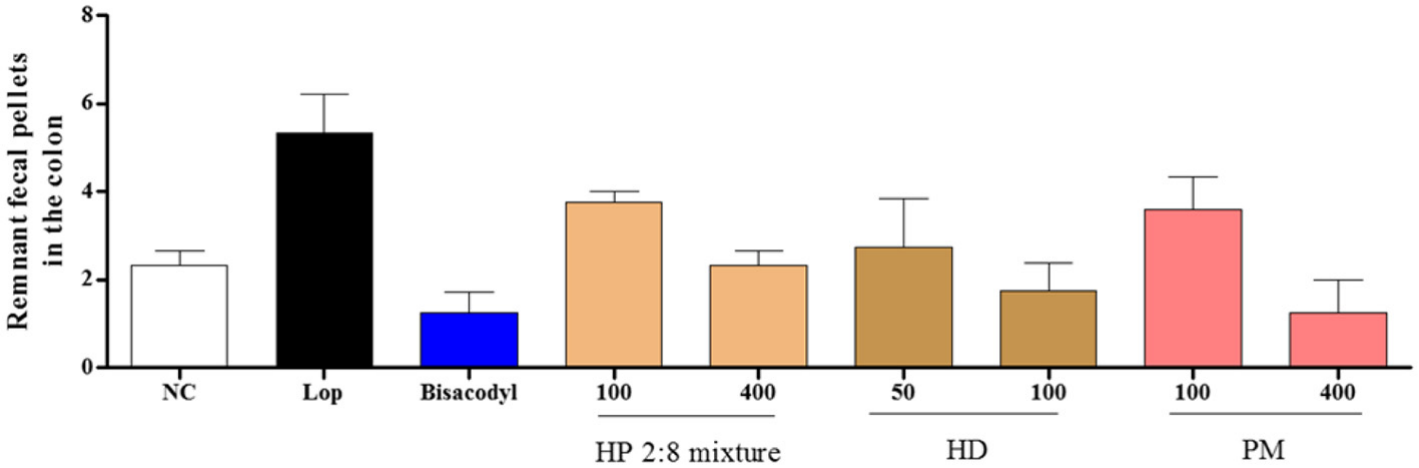
Effect of *Hovenia dulcis* Thunb. (HD) and *Phyllostachys pubescens* Mazel (PM) mixed (2:8) hot-water extract (HP 2:8 mixture) on remnant fecal pellets in the colon in rats with loperamide-induced constipation. The values are expressed as the mean ± SEM. (*n* = 7, *p < 0.05, **p < 0.01, ***p < 0.001 significantly different from the loperamide group) Statistical significance was tested with post hoc Dunnett test. NC: normal control, Lop: loperamide treated group, Bisacodyl: positive control, HP: *Hovenia dulcis* Thunb. (HD) and *Phyllostachys pubescens* Mazel (PM) 2:8 mixture.

Gastrointestinal Charcoal Transit ratio and Distal Colon Fecal Number in Loperamide-Induced Constipation To determine the effects of HP mixed extracts on the intestinal transit ratio, we measured gastrointestinal charcoal transit ratio in the rat. The intestinal charcoal transit ratio was significantly reduced in the loperamide group compared to that of the normal control group, and enhanced in the positive control group and all sample treatment groups (Fig. 4). Fecal numbers in the distal colon increased in the loperamide group compared to the normal control group and were decreased in all sample treatment groups compared to the loperamide group, but there was no significant difference from the loperamide group (Fig. 5).

### Gastrointestinal Charcoal Transit Ratio and Distal Colon Fecal Number in Low-Fiber Diet-Induced Constipation

**Fig. 6:**
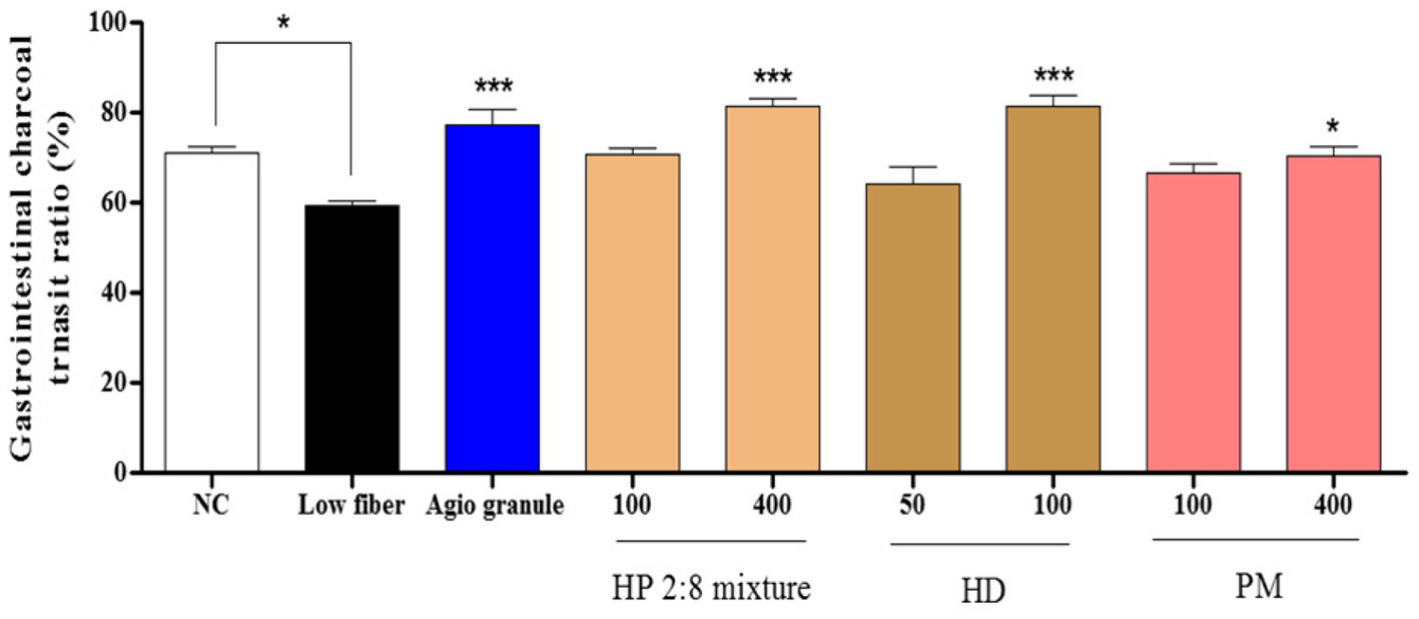
Effect of *Hovenia dulcis* Thunb. (HD) and *Phyllostachys pubescens* Mazel (PM) mixed (2:8) hot-water extract (HP 2:8 mixture) on gastrointestinal charcoal transit ratio in rats with low-fiber diet-induced constipation. The values are expressed as the mean ± SEM (*n* = 7, *p < 0.05, **p < 0.01, ***p < 0.001 significantly different from the low-fiber diet group). Statistical significance was tested with post hoc Dunnett test. NC: normal control, Low-fiber: low-fiber diet group, Agio granule: positive control, HP: *Hovenia dulcis* Thunb. (HD) and *Phyllostachys pubescens* Mazel (PM) 2:8 mixture.

**Fig. 7:**
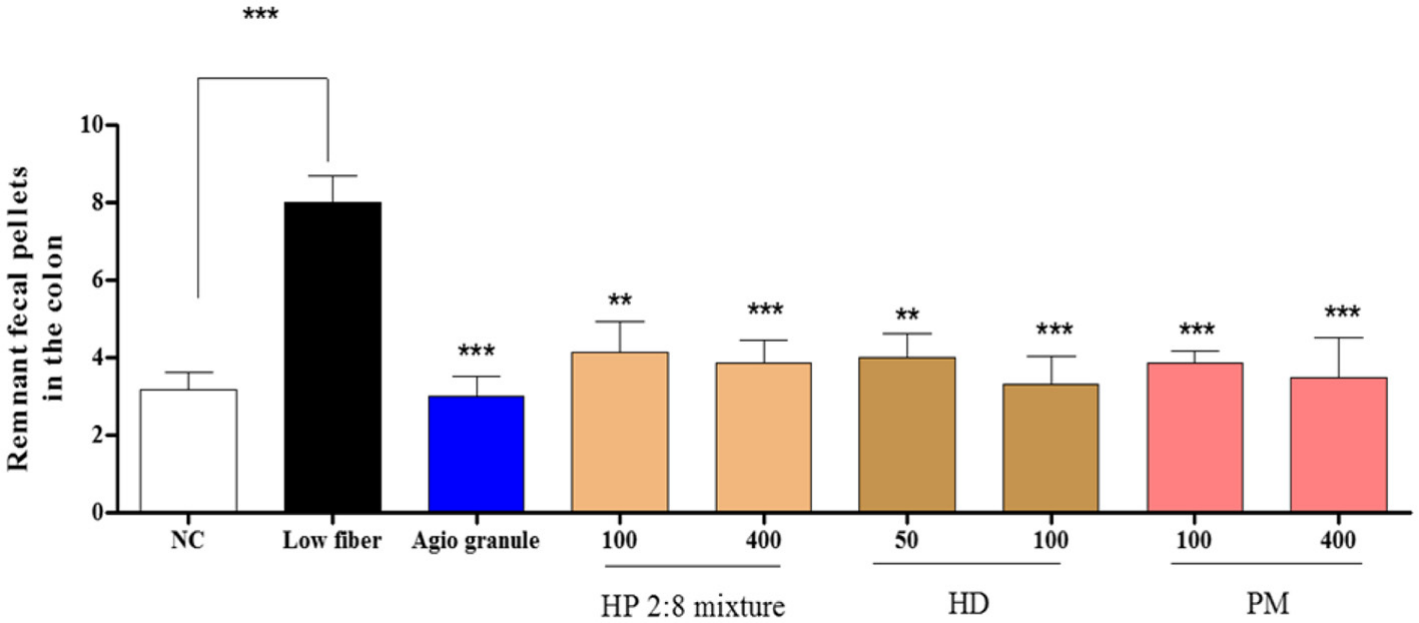
Effect of *Hovenia dulcis* Thunb. (HD) and *Phyllostachys pubescens* Mazel (PM) mixed (2:8) hot-water extract (HP 2:8 mixture) on remnant fecal pellets in the colon in rats with low-fiber diet-induced constipation. The values are expressed as the mean ± SEM (*n* = 7, *p < 0.05, **p < 0.01, ***p < 0.001 significantly different from the low-fiber diet group). Statistical significance was tested with post hoc Dunnett test. NC: normal control, Low-fiber: low-fiber diet group, Agio granule: positive control, HP: *Hovenia dulcis* Thunb. (HD) and *Phyllostachys pubescens* Mazel (PM) 2:8 mixture.

To determine the effects of HP mixed extracts on the intestinal transit ratio in low-fiber diet-induced constipation, we measured gastrointestinal charcoal transit ratio in the rat. Intestinal charcoal transit ratio was reduced in the loperamide group compared to that of the normal control group, and enhanced in the positive control group and high-dose treatment groups (Fig. 6). Fecal numbers in the distal colon increased in the loperamide group compared to those of the normal control group and were decreased in all sample treatment groups compared those of the loperamide group (Fig. 7).

### Histology of the Distal Colon in Loperamide-Induced Constipation

**Fig. 8:**
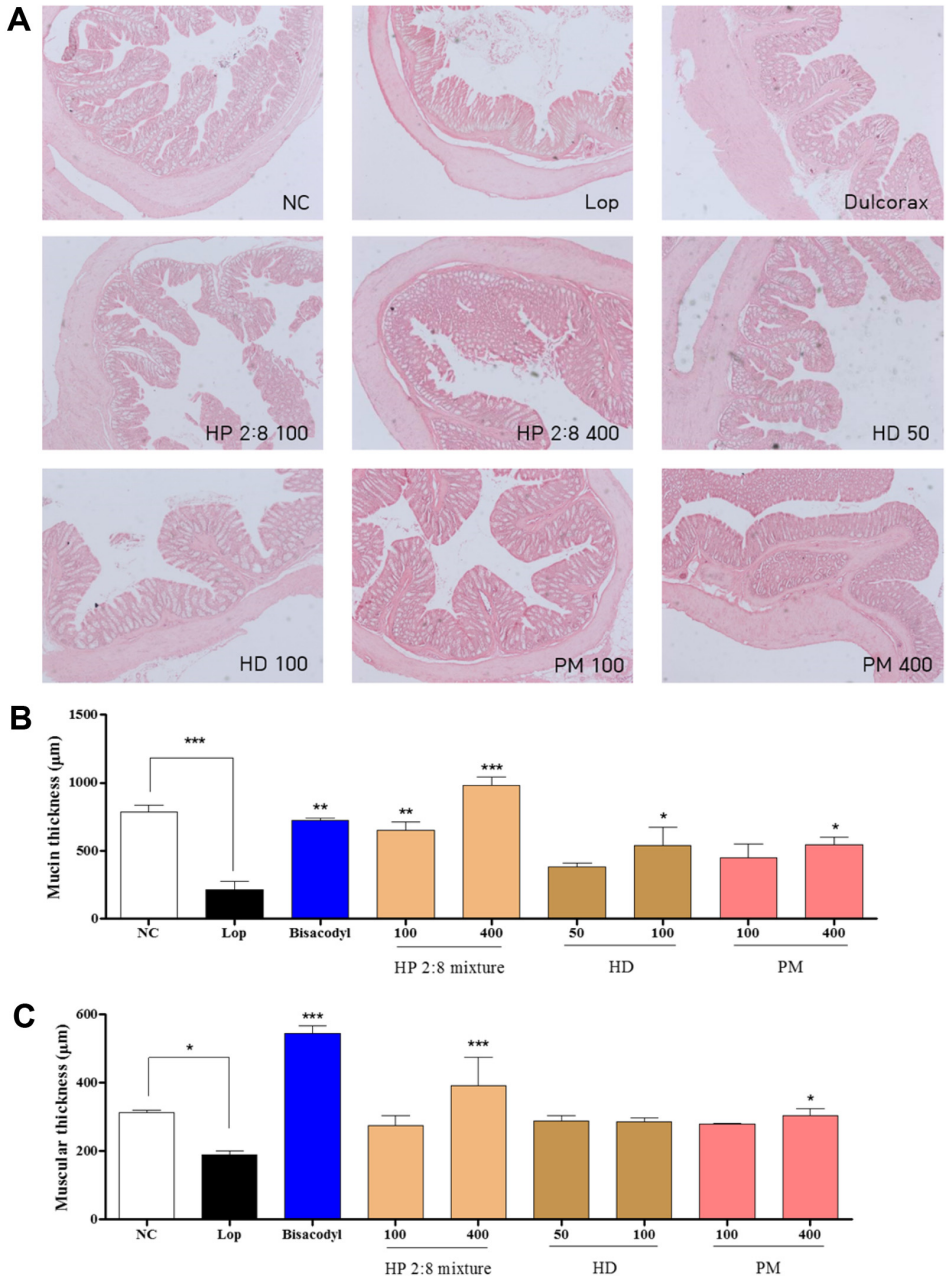
Alteration of the histological structure in the colon. (**A**) Hematoxylin and eosin (H&E) stained sections of colons from the untreated group, loperamide group, positive group, the HP 2:8 mixture group (100 and 400 mg/kg), HD-only treated group (50 and 100 mg/kg), and PM-only treated group (100 and 400 mg/kg) were observed at 50× magnification using a light microscope. (**B**) The thickness of the mucosa and (**C**) muscular layer are presented as graphs. The values are expressed as the mean ± SEM (*n* = 7, *p < 0.05, **p < 0.01, ***p < 0.001 significantly different from the loperamide group). Statistical significance was tested with post hoc Dunnett test. NC: normal control, Lop: loperamide treated group, Bisacodyl: positive control, HP: *Hovenia dulcis* Thunb. (HD) and *Phyllostachys pubescens* Mazel (PM) 2:8 mixture.

To investigate whether HP mixed extracts treatment could induce alteration of the histological structure of the distal colon in loperamide-induced constipation, mucosa layer thickness and muscle thickness were assessed in the distal colons of rats following H&E staining. The average thickness of mucosa was significantly less in the loperamide group than the normal control group. Following treatment of the loperamide-induced constipation with HP mixed extracts (100 and 400 mg/kg), this level increased by more than 67.11% and 78.14% compared with that of the loperamide group. This level also increased in the HD-only treatment (50 and 100 mg/kg) and PM-only treatment (100 and 400 mg/kg) groups by 43.67%, 60.38%, 52.02%, and 60.72% respectively (Figs. 8A and 8B). Moreover, muscle thickness was very similar to mucosa layer thickness. In the loperamide group, the muscle thickness was dramatically decreased compared to that of the normal control group. However, with the HP mixed extracts treatment (12.68% and 37.77%), HD-only treatment (23.96% and 24.81%), and PM-only treatment (22.02% and 27.64%) with loperamide groups, muscle thickness was recovered (Fig. 8C). Taken together, these results show that HP mixed extracts induced increases in mucosa layer and muscle thickness in the distal colon of constipated rats.

### Histology of the Distal Colon in Low-Fiber Diet-Induced Constipation

**Fig. 9:**
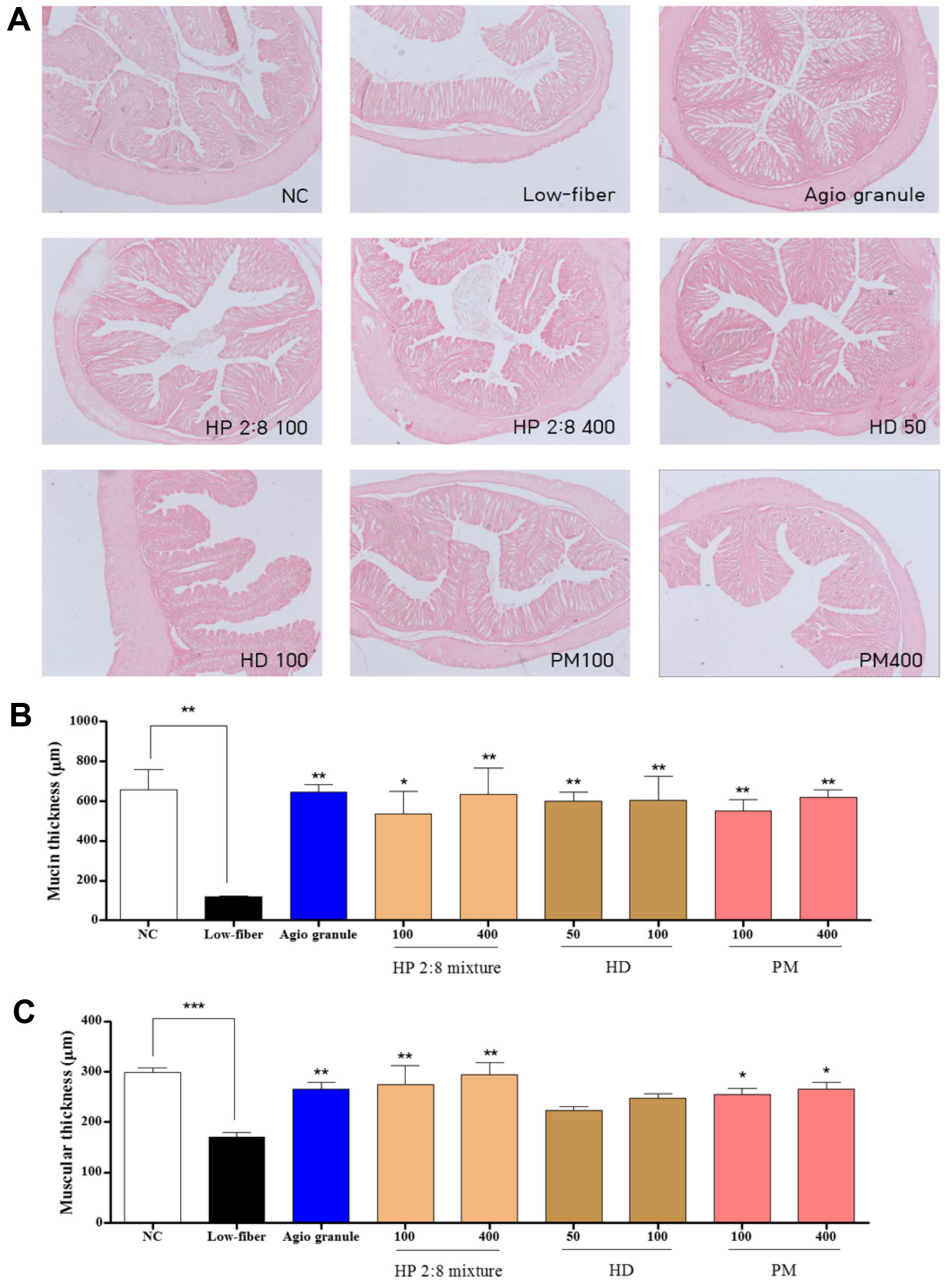
Alteration of the histological structure in the colons. (**A**) Hematoxylin and eosin (H&E) stained sections of colons from the untreated group, low-fiber diet group, positive group, the HP 2:8 mixture group (100 and 400 mg/kg), HD-only treated group (50 and 100 mg/kg), and PM-only treated group (100 and 400 mg/kg) were observed at 50× magnification using a light microscope. (**B**) The thickness of the mucosa and (**C**) muscular layer are presented as graphs. The values are expressed as the mean ± SEM (*n* = 7, *p < 0.05, **p < 0.01, ***p < 0.001 significantly different from the low-fiber diet group). Statistical significance was tested with post hoc Dunnett test. NC: normal control, Low-fiber: low-fiber diet group, Agio granule: positive control, HP: *Hovenia dulcis* Thunb. (HD) and *Phyllostachys pubescens* Mazel (PM) 2:8 mixture.

To investigate whether HP mixed extracts treatment could induce alteration of the histological structure of the distal colon in low-fiber diet-induced constipation, mucosa layer thickness, and muscle thickness were assessed in the distal colons of rats following H&E staining. The average thickness of mucosa was significantly lower in the low-fiber diet group than that of the normal control group. Following HP mixed extracts treatment for the low-fiber diet (100 and 400 mg/kg), this level increased by more than 78.05% and 81.40% compared with that of the low-fiber diet group. This level also increased in HD-only treatment (50 and 100 mg/kg) and PM-only treatment (100 and 400 mg/kg) group by 80.31%, 80.47%, 78.69%, and 80.94% respectively (Figs. 9A and 9B). Moreover, muscle thickness was very similar to mucosa layer thickness. In the low-fiber diet group, the muscle thickness was dramatically decreased compared to that of the normal control group. However, in the HP mixed extracts treatment (34.61% and 36373%), HD-only treatment (18.76% and 24.38%), and PM-only treatment (26.65% and 29.26%) with low-fiber diet groups, muscle thickness was recovered (Fig. 9C). Taken together, these results show that HP mixed extracts induced increases in mucosa layer and muscle thickness in the distal colon of constipated rats.

### Effects of the HP 2:8 Mixture on Spontaneous Colon Contraction

**Fig. 10:**
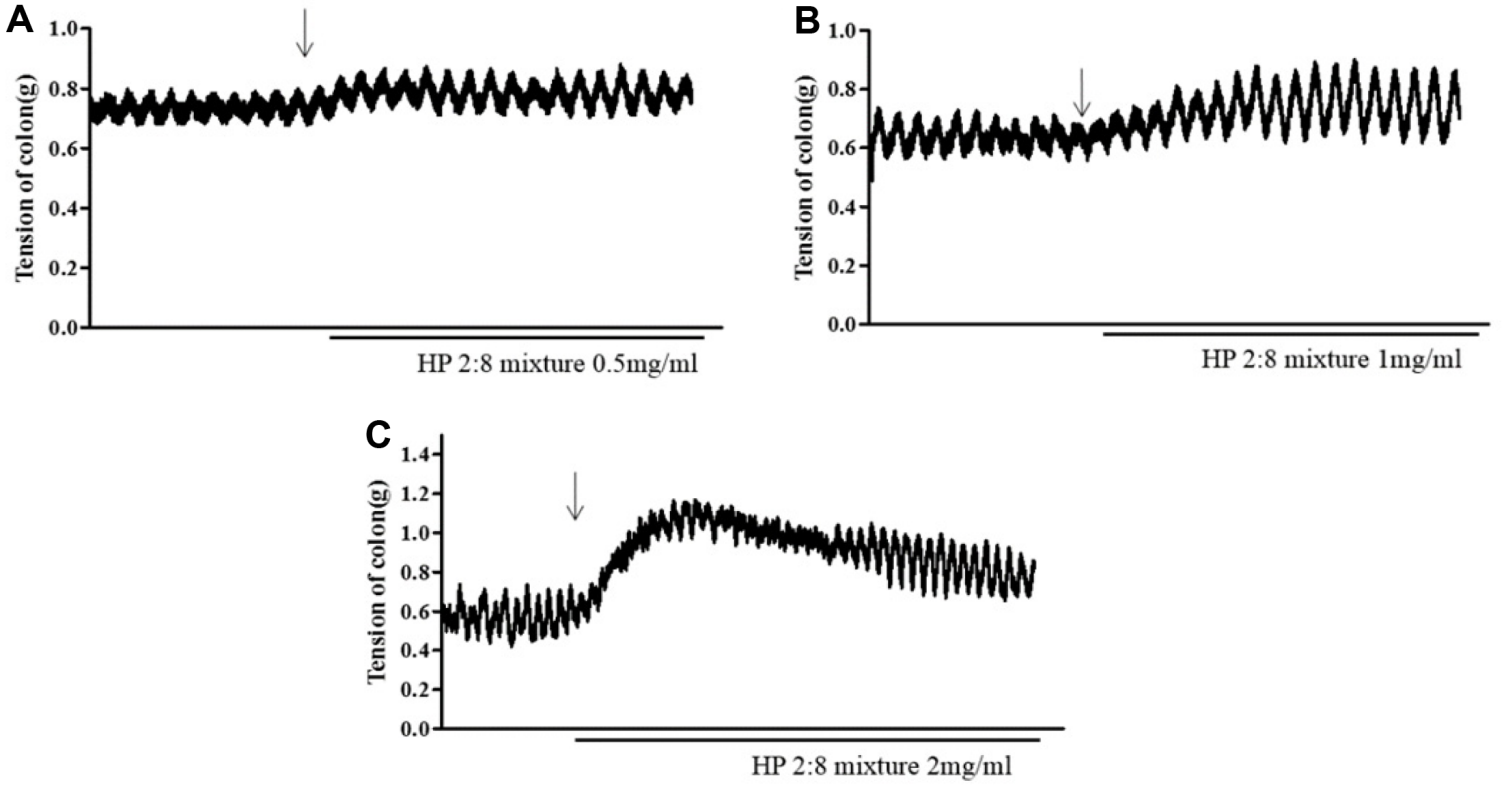
Representative temporal profiles of contractile activity of isolated rat colon in the *Hovenia dulcis* Thunb. (HD) and *Phyllostachys pubescens* Mazel (PM) mixed (2:8) hot-water extract (HP 2:8 mixture) at 0.5 mg/ml (**A**), 1 mg/ml (**B**), and 2 mg/ml (**C**).

To investigate the effect of *Hovenia dulcis* Thunb. (HD) and *Phyllostachys pubescens* Mazel (PM) mixed (2:8) hot-water extract (HP 2:8 mixture) on spontaneous intestinal contraction, we assessed the tension of the isolated rat colon segments. The HP 2:8 mixture was applied to the isolated colon at final concentrations of 0.5, 1, and 2 mg/ml. In the isolated intestinal colon, tension of the intestinal colon was significantly increased in a dose-dependent manner (Fig. 10).

### Effects of HP 2:8 Mixture on IEC-18 Cell [Ca^2+^]i

**Fig. 11:**
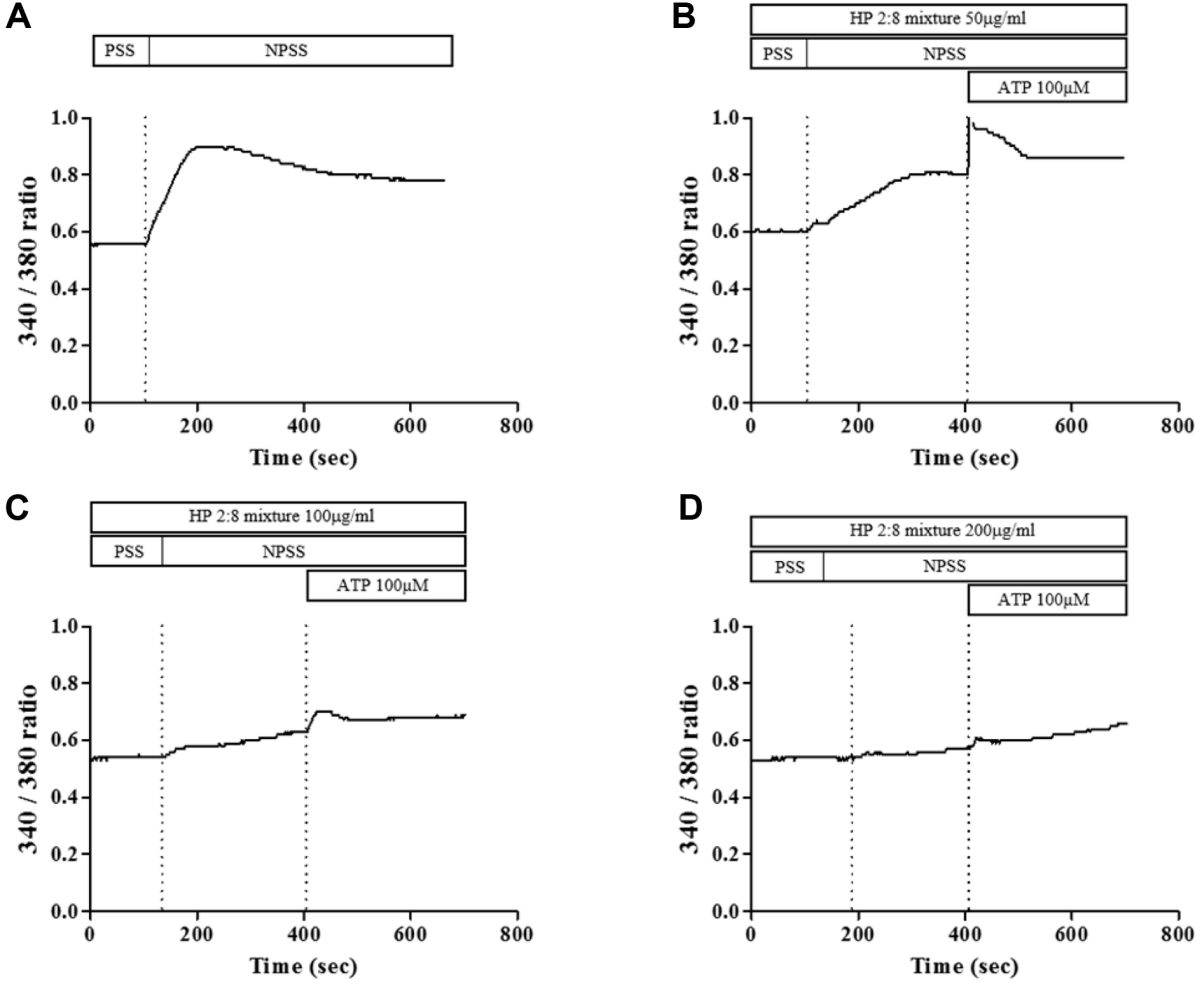
Effect of HP 2:8 mixture on [Ca^2+^]i of IEC-18 cells. (**A**) Ca^2+^ influx by changing the buffer from Ca^2+^-free (PSS) to normal solution (NPSS) induced a dramatic increase in [Ca^2+^]i detected by fura-2/AM. (**B**) ATP-induced Ca^2+^ release was blocked in the presence of HP 2:8 mixture 50mg/ml. (**C**) ATP-induced Ca^2+^ release was blocked in the presence of HP 2:8 mixture 100 mg/ml. (**D**) ATP-induced Ca^2+^ release was blocked in the presence of HP 2:8 mixture 200 mg/ml. All the data are represented as means ± SEM.

To define the cellular mechanisms that mediate HP 2:8 mixture-induced relaxation, we used an intestinal epithelial cell line. A fluorescence signal increase was initiated when the bath solution was changed from Ca^2+^ free PSS to a normal PSS (Fig. 11A); while this increase was not observed in the presence of HP 2:8 mixture when Ca^2+^ influx was blocked. The HP 2:8 mixture could also prevent the ATP induced [Ca^2+^]i increase (Figs. 11B-11D).

Constipation is a common health problem that tends to cause discomfort and affect patient quality of life [36]. Constipation may cause abdominal bloating, sickness, stress, and digestive problems [37]. Currently, a variety of therapeutic approaches are used for constipation. Treatments for constipation usually include stimulant and osmotic laxatives, fiber supplements, fecal softeners, and sometimes enemas for wayward constipation. As current symptomatic treatments can produce unsatisfactory results, many patients seek help from herbal medicine [38]. Any herbal medicine may contain many bioactive compounds with potentially deleterious as well as beneficial effects [39].

In the present study, we investigated the laxative effects of the mixture of *Hovenia dulcis* Thunb. and *Phyllostachys pubescens* Manzel (HP 2:8 mixture) on chronic constipation. Loperamide-induced and low-fiber diet-induced constipation are well-established methods that are widely used as a model of spastic constipation [40]. In this study the results demonstrated that HP mixed extract can elevate the symptoms of constipation through the improvement of fecal excretion, and the recovery of histological changes of the colon in a constipation model.

The most important factor during the mechanistic and developmental study of laxative drugs is fecal excretion. The constipation model induced with loperamide and a low-fiber diet showed a significant decrease in fecal parameters including feces weight, number, and water consumption [41-43]. These changes were alleviated with several plant extracts. In this study, a similar laxative effect on fecal parameters was verified in the loperamide and low-fiber diet-induced constipation model by administration of 100 or 400 mg/kg of HP mixed extract, 50 or 100 mg/kg of *Hovenia dulcis* Thunb. (HD), and 100 or 400 mg/kg of *Phyllostachys pubescens* Manzel (PM) (Figs. 2 and 3). These results provide evidence that HP mixed extract can contribute to alleviating constipation, and are potential candidates for laxative drugs.

The intestinal transit ratio has most often been measured by administering an aqueous suspension of active charcoal to rodents, and transit ratio is reflective of intestinal motility. Motility of the gastrointestinal (GI) tract, evaluated by the charcoal meal, indicated the rapid and significant excretion of fecals in response to treatment with the HP 2:8 mixture compared with that of the loperamide-induced group and low-fiber diet-induced group. The GI tract is regulated by the innervation of the enteric nervous system (ENS), which controls the rhythmic activity of the gut smooth muscle cells [44]. The HP 2:8 mixture at a dose of 100 and 400 mg/kg significantly increased the distance traveled by charcoal meal in loperamide-induced constipation (Figs. 4 and 5). Also, HP mixed extract at a dose of 400 mg/kg increased the transit ratio in low-fiber diet-induced constipation (Figs. 6 and 7).

Mucin is responsible for the physical and chemical properties of mucus [14]. Colonic mucus is decreased in a rat model of constipation, and mucosal layer thickness in the distal colon of rats is decreased by loperamide [45] and a low-fiber diet [46]. In this study, the mucosa layer length and muscular layer thickness sharply increased in the groups with the HP 2:8 mixture at 100 and 400 mg/kg compared with those of the loperamide-treated group (Fig. 8). It has same result in the low-fiber diet-induced constipation model (Fig. 9).

The coordination of smooth muscle contraction and relaxation is the basis of gut motility [47]. Modern pharmacological agents including senna and bisacodyl are generally used for the treatment of constipation and these agents can increase the contraction of intestinal smooth muscle [48]. The spontaneous contractions in isolated rat colon were enhanced by treatment with to 0.5, 1, and 2 mg/ml of HP 2:8 mixture (Fig. 10). The significant increase in contraction suggests that HP mixed extract has a laxative effect and could be efficacious for the treatment of constipation. We also confirmed effect of single samples (HD and PM) on spontaneous intestinal contraction. HD and PM has effect of contractile activity but it was insignificant effect then HP 2:8 mixture (data not shown).

It has been reported that ATP released from enteric nerves mediates muscular inhibitory and excitatory responses in various animal species via activation of P2X and/or P2Y receptors [49]. ATP has been reported to induce inhibitory effects in all area of the gut acting on P2Y receptors [50-52], while excitatory effects on count of activation of P2X receptors placed on excitatory nerves in the stomach [53] or on smooth muscle in the longitudinal muscle of the colon have been shown [50]. The contraction effects induced by ATP were antagonized by the suramine (ATP inhibitor) or 4-[[4-formyl-5-hydroxy-6-methyl-3-[(phosphonooxy)methyl]-2-pyridinyl]azo]-1,3-benzene disulphonic acid (PPADS) [54]. Also, mobilization of Ca^2+^ and an increase in [Ca^2+^]i are required to trigger smooth muscle contraction [55]. ATP is a molecule known to produce Ca^2+^ responses in astrocytes, and has been implicated as a mediator of intercellular Ca^2+^ signaling in other types of non-excitable cells [56].

In the attempt to characterize the ATP response, we tested ATP-induced contractions in the presence of HP 2:8 mixture on NPSS. Fig. 10 showed that HP 2:8 mixture had an inhibitory effect on ATP-mediated external Ca^2+^ -evoked contractions (Fig. 11).

Taken together, the results of this study suggest that HP 2:8 mixture induces the recovery of fecal excretion, gastrointestinal motility, and histopathology in loperamide-induced and low-fiber diet-induced constipation models. Indeed, [Ca^2+^]i measurements by using fura-2 as an indicator showed HP 2:8 mixture has inhibitory responses in rat colon by inhibiting ATP-mediated Ca^2+^ influx. Therefore, our findings are demonstrate that the mixture of *Hovenia dulcis* Thunb. and *Phyllostachys pubescens* Manzel is a useful therapeutic option for the treatment of constipation.

In this experiment, we investigated the effect of the mixed extract of *Hovenia dulcis* Thunb. (HD) and *Phyllostachys pubescens* Mazel (PM) on chronic constipation in two constipation animal models and tried to clarify the mechanism of this effect. As a result, it was confirmed that the constipation improvement parameters (fecal weight, fecal number, and fecal moisture content) were improved in both the loperamide-induced animal model and the low-diet-induced animal model. Histological structure of the distal colon revealed that the mucin layer and muscular layer thickness decreased in the groups where constipation was induced (loperamide and low-fiber diet group) and increased in the HP 2:8 mixture treatment group. In addition, the contractile activity of colon tissues in the HP 2:8 mixture was found to increase with concentration. Moreover, the HP 2:8 mixture has inhibitory responses in rat colon, by inhibiting ATP-mediated Ca^2+^ influx. From these results, it was confirmed that the HP 2:8 mixture in the constipation model smooths mucin secretion and stimulates intestinal motility, thereby laxative constipation.

## Figures and Tables

**Table 1:** Composition of normal diet and low-fiber diet.

Ingredients	Contents (%)	

	Normal diet	Low-fiber diet
Moisture	9.2	9
Fiber	4.7	0.1
Protein	20	21.9
Fat	9.9	6.1
Ash	6	5.9
Nitrogen-free extract	50.2	57

**Table 2:** Effects of Hovenia dulcis Thunb. (HD) and Phyllostachys pubescens Mazel (PM) mixed (2:8) hot-water extract (HP 2:8 mixture) on body weight, food intake and water intake in loperamide-induced constipation.

	Body weight (g/day)			Food intake (g/day)		Water intake (ml/day)	

	Initial	Final	Body weight gain	Before constipation	After constipation	Before constipation	After constipation
Normal control	157.45±8.13	260.77±14.96	103.32±12.19	24.44±1.95	26.01±2.02	34.43±4.54	32.14±6.99
Loperamide 4 mg/kg	157.56±8.01	229.71±7.72	72.15±9.24^***^	23.29±1.20	22.65±1.29	31.71±3.15	30.71±4.50
Bisacodyl 4 mg/kg	157.33±7.45	237.25±14.08	79.92±13.91^**^	21.88±2.45	20.76±1.54	31.29±5.09	32.14±4.38
HP 100 mg/kg	157.38±7.28	235.8±15.36	78.42±13.07^**^	22.72±1.64	21.99±2.09	34.29±5.56	30.71±7.35
HP 400 mg/kg	157.36±7.44	234.81±13.83	77.46±9.54^***^	21.42±0.84	21.05±2.61	31.00±5.32	27.14±5.67
HD 50 mg/kg	157.43±7.47	235.1±12.49	77.67±14.24^**^	22.46±1.10	20.84±1.95	32.87±5.58	29.29±7.32
HD 100 mg/kg	157.45±7.54	238.97±14.95	81.53±9.23^**^	22.34±1.60	20.54±5.29	32.57±4.89	35.28±5.16
PM 100 mg/kg	157.14±7.66	237.43±16.22	80.29±13.34^**^	22.10±2.41	20.90±1.64	32.00±5.29	32.86±4.88
PM 400 mg/kg	157.39±8.34	230.42±16.46	73.03±11.57^***^	22.37±1.93	19.54±6.91	34.43±3.56	35.57±4.08

Data represent the mean ± SEM. Values with different letters in a column are significantly differnet according to Student’s *t*-test (*p* < 0.05). ^*^p < 0.05, ^**^p < 0.01, ^***^p < 0.001 significantly different from the normal control group.

**Table 3:** Effects of Hovenia dulcis Thunb. (HD) and Phyllostachys pubescens Mazel (PM) mixed (2:8) hot-water extract (HP 2:8 mixture) on body weight, food intake, and water intake in low-fiber diet-induced constipation.

	Body weight (g/day)			Food intake (g/day)		Water intake (ml/day)	

	Initial	Final	Body weight gain	Before constipation	After constipation	Before constipation	After constipation
Normal control	257.95±10.01	363.63±11.30	105.68±10.03	21.85±2.16	30.07±1.80	32.43±3.82	38.29±2.36
Low-fiber diet	263.59±5.99	390.46±23.15	126.87±16.93	17.72±2.56	24.64±0.67	28.57±4.76	29.71±4.15
Agio granulel 620 mg/kg	263.24±5.95	383.65±17.81	120.41±11.98	18.70±1.73	24.27±0.47	27.86±3.93	30.29±2.98
HP 100 mg/kg	259.24±11.90	383.19±24.34	123.95±8.62	17.34±3.45	23.94±1.47	24.29±4.50	29.29±3.45
HP 400 mg/kg	259.55±8.11	383.65±28.35	124.10±20.80	18.16±3.32	24.02±0.54	23.57±9.00	28.14±4.18
HD 50 mg/kg	271.56±12.63	407.39±34.66	135.83±43.29	17.82±3.41	24.84±0.80	27.86±6.99	28.86±3.98
HD 100 mg/kg	256.81±9.91	376.00±28.73	119.19±33.38	21.21±1.54	24.47±0.84	23.00±4.65	28.86±2.73
PM 100 mg/kg	258.10±10.00	380.40±22.51	122.30±11.05	20.90±2.64	24.16±0.69	24.29±6.07	31.00±1.91
PM 400 mg/kg	265.91±5.26	393.84±19.11	127.93±13.03	18.88±1.84	24.32±1.40	27.86±2.67	29.86±2.34

Data represent the mean ± SEM. Values with different letters in a column are significantly differnet according to Student’s t-test (*p* < 0.05). ^*^p < 0.05, ^**^p < 0.01, ^***^p < 0.001 significantly different from the normal control group.
